# Role of Re-entry Tears on the Dynamics of Type B Dissection Flap

**DOI:** 10.1007/s10439-017-1940-3

**Published:** 2017-10-30

**Authors:** Saranya Canchi, Xiaomei Guo, Matt Phillips, Zachary Berwick, Jarin Kratzberg, Joshua Krieger, Blayne Roeder, Stephan Haulon, Sean Chambers, Ghassan S. Kassab

**Affiliations:** 1 California Medical Innovations Institute, 11107 Roselle St., Rm. 211, San Diego, CA 92121 USA; 2 3DT holdings LLC, San Diego, CA USA; 3 Cook Medical Inc, Bloomington, IN USA; 4 Aortic Center, Hôpital Cardiologique, CHU de Lille, Lille, France

**Keywords:** Acute aortic dissection, *Ex vivo* model, Clinical FDR, Lumen pressure, Dilation, Strain

## Abstract

Mortality during follow-up after acute Type B aortic dissection is substantial with aortic expansion observed in over 59% of the patients. Lumen pressure differential is considered a prime contributing factor for aortic dilation after propagation. The objective of the study was to evaluate the relationship between changes in vessel geometry with and without lumen pressure differential post propagation in an *ex vivo* porcine model with comparison with patient clinical data. A pulse duplicator system was utilized to propagate the dissection within descending thoracic porcine aortic vessels for set proximal (%circumference of the entry tear: 40%, axial length: 2 cm) and re-entry (50% of distal vessel circumference) tear geometry. Measurements of lumen pressure differential were made along with quantification of vessel geometry (*n* = 16). The magnitude of mean lumen pressure difference measured after propagation was low (~ 5 mmHg) with higher pressures measured in false lumen and as anticipated the pressure difference approached zero after the creation of distal re-entry tear. False lumen Dissection Ratio (FDR) defined as arc length of dissected wall divided by arc length of dissection flap, had mean value of 1.59 ± 0.01 at pressure of 120/80 mmHg post propagation with increasing values with increase in pulse pressure that was not rescued with the creation of distal re-entry tear (*p* < 0.01). An average FDR of 1.87 ± 0.27 was measured in patients with acute Type B dissection. Higher FDR value (FDR = 1 implies zero dissection) in the presence of distal re-entry tear demonstrates an acute change in vessel morphology in response to the dissection independent of local pressure changes challenges the re-apposition of the aortic wall.

## Introduction

The current treatment for Type B dissection which always includes best medical, treatment, and eventually endovascular or surgical intervention or a combination of both, aims to stabilize the hemodynamics and restore blood flow to end organs, and reduce the risk of rupture. Early mortality from Type B aortic dissection is substantial, varying between 10 and 29%^24^,^29^,^32^ with follow-up mortality rates approaching 1 in every 4 patients at 3 years.^34^ Aortic expansion during follow-up was reported in 59% of patients with average rate of 1.7 ± 0.7 mm/year.^5^,^13^ Computed tomography show the acute enlargement of dissected aorta of almost 25% from its baseline.^23^

Currently, the risk factor to predict aortic dissection dilation is the aortic diameter. The criteria for repair of aneurysmal dissection are a diameter > 5.0 to 6.0 cm, or rapid expansion in a chronic aneurysmal dissection > 1 cm/year.^8^,^30^ In a clinical study^27^ performed across 101 patients with Type B acute dissection without complications, a maximum aortic diameter of > 4 cm and a patent false lumen during the acute phase served as important predictors for aortic enlargement in the chronic phase.

Although several *in vivo* and *ex vivo* studies have been reported describing the patterns of dissection and the associated characteristics of hemodynamic parameters, flow and velocity,^2^,^3^,^6^,^9^,^21^,^31^ few studies have examined the lumen pressure differential and the risk for aortic dilation. Phantom *ex vivo* models using polymer tubes (which have different physiological properties than biological tissue and a rigid flap) have been used to show the relationship between tear configuration (size, number, location) and false lumen pressure.^1^,^25^,^35^ The shape of the pressure waveforms in both the lumens were shown to be identical in an artificially created dissection in an *ex vivo* porcine model.^21^ An *in vivo* study using a swine model of retrograde endovascular aortic dissection with re-entry tear report aortic dilation at the dissection portion.^19^ The depth of the dissection plain was, however, unpredictable, inconsistent and technique dependent.

To systematically understand the spontaneous antegrade Type B dissection, we have recently shown the relationship between initial tear geometry and pulse pressure magnitude for initiation and propagation of dissection.^20^ Using an *ex vivo* porcine model for spontaneous Type B dissection we reproduce the lumen pressure characteristics with and without distal re-entry tear, evaluate the changes in the dissected vessel geometry and compare against measurements made in aortae of patients with acute Type B dissection.

## Methods

### Tissue Sample Preparation

The descending thoracic porcine aortas were obtained from local slaughterhouse (Sierra for medical science, Whittier, CA, USA) and cut to 20 cm in length from the arch. Fresh samples were stored in 0.9% saline solution followed by preparation and testing within 24 h of tissue arrival at the facility. The aortae were prepared for testing by removing any loose adventitial tissue and ligating the major branches using silk suture (Henry Schien, Melville, NJ, USA), as deemed necessary.

#### Initial Entry Tear

All initial entry tears were manually created inside the vessel, at ~ 5 cm from the arch using a surgical blade. The %circumferential length of the entry tear defined previously in Peelukhana *et al.* 20 was created along the vessel circumference and was set at 40%. The dissection plane was set in the media layer at 1/3rd the vessel wall thickness. The layers were separated and advanced using fine-tip forceps to the desired axial length of 2 cm (measured along the length of the vessel) and later confirmed using ultrasound (US) measurements. This created the true lumen (TL) and false lumen (FL) within the vessel.

#### Re-entry Tear

The aorta was flipped inside out after exposure to the pulsatile flow. The final tear circumference at proximal end and the axial length of the propagated flap were measured using a suture and a ruler respectively. A re-entry tear was created at the distal end of the propagated flap by using a surgical blade. The %circumferential length of the re-entry tear was set at 50% of distal vessel circumference.

### Pulse Duplicator Test System

**Figure 1:**
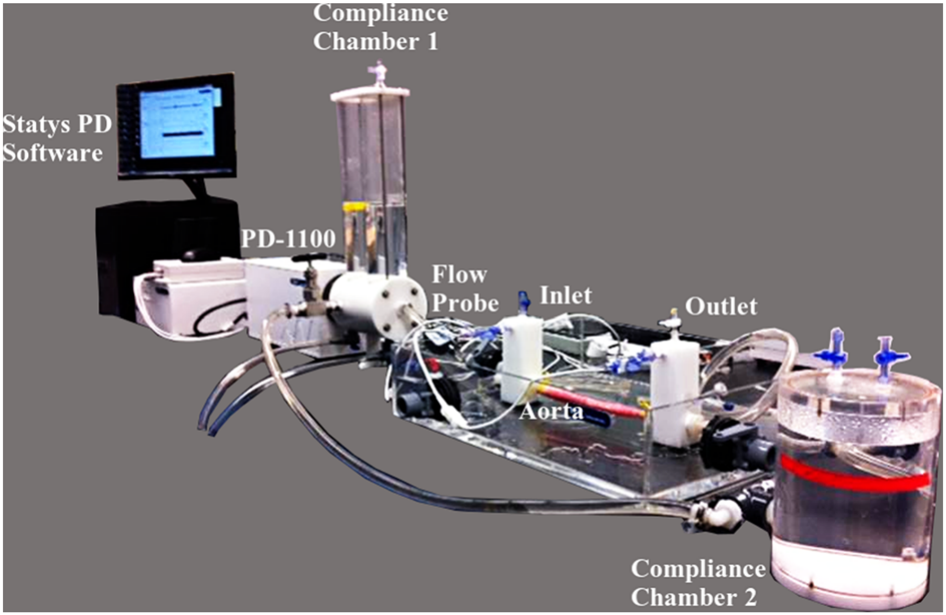
Experimental set-up showing the major components. PD-1100 is the pulsatile flow pump, and is attached to the compliance chamber 1 (CC1). The outlet from CC1 is connected to a saline tank that houses the inlet and outlet ports to mount the aorta. CC2 acts as a downstream capacitor and the flow loops back to the CC1. Integrated Statys PD software is used for data acquisition.

A pulse duplicator [PD] (PD; BDC laboratories, Wheat Ridge, CO, USA) reproduced the physiological flow and pressures in the porcine aorta vessel integrated within the test system (Fig. 1). Detailed description of the setup can be found in Peelukhana *et al.* 20 Inlet flow rate was measured using an inline flow probe (ME13PXN; Transonics Inc., Ithaca, NY, USA) was set to 2 L/min for all tested aorta samples. The pump parameters were set constant for each experiment (HR: 72 bpm, Systole/diastole ratio = 35/65%). Based on previous porcine *in vivo* length measurement of the aorta, an average stretch ratio of 30% was incorporated for all the vessels prior to testing by adjusting the distance between the inlet and outlet ports.

CIRS blood mimicking fluid was used as blood analog, with a *ρ* = 1050 kg/m^3^, and dynamic viscosity of *μ* = 4 cP or 0.004 Pa.s (Model 046, Computerized Imaging Reference Systems, Incorporated (CIRS), Norfolk, VA, USA). The container tank was filled with 0.9% saline solution such that the test vessel was completely submerged.

The inlet pressure was set at 120/80 mmHg. The pulse pressure was subsequently increased in steps of 20 mmHg. The test vessel was subjected to each pressure step for 3 min before the next pressure increment. This stepwise increase continued till propagation occurred and/or the limit of the PD system was reached which was set at 300 mmHg. Any dissection that propagated above pulse pressure of ~ 200 mmHg was not included for data analysis.

### Data Acquisition

To obtain the local pressures within the FL and TL, 7 Fr. catheters were enabled within the inlet and outlet ports, respectively. The pressure and flow data was obtained for 5 s at 5000 Hz frequency using Statys PD integrated data-acquisition system. A L15-i70 ultrasound (US) transducer connected to IE33 ultrasound machine (Phillips Ultrasound, Bothell, WA, USA) was used for visualization. The PD pump output trigger was connected US ECG leads to synchronize the pressure from the Statys PD software with the flap movement.

Prior to start of the experiment, the TL and FL pressure catheters were adjusted in the proximal end of the entry tear and confirmed using the Echo imaging. The catheters were held for 3 min at each location prior to measurement to allow for the pressure to equilibrate. The velocities were measured in the pulse wave Doppler mode while flap and lumen geometric characteristics were imaged in B-mode.

Ten porcine aorta vessels were used to obtain the lumen pressure differential. The methodology without the catheters was replicated to obtain geometric relationship between false lumen and flap (*n* = 6). The delta lumen pressure difference was calculated by subtracting the TL from the FL pressures after propagation with and without a single distal re-entry tear. For a given test vessel, the average time of exposure to the pulsatile flow was 1.5 ± 0.3 h.

### Data Analysis

**Figure 2:**
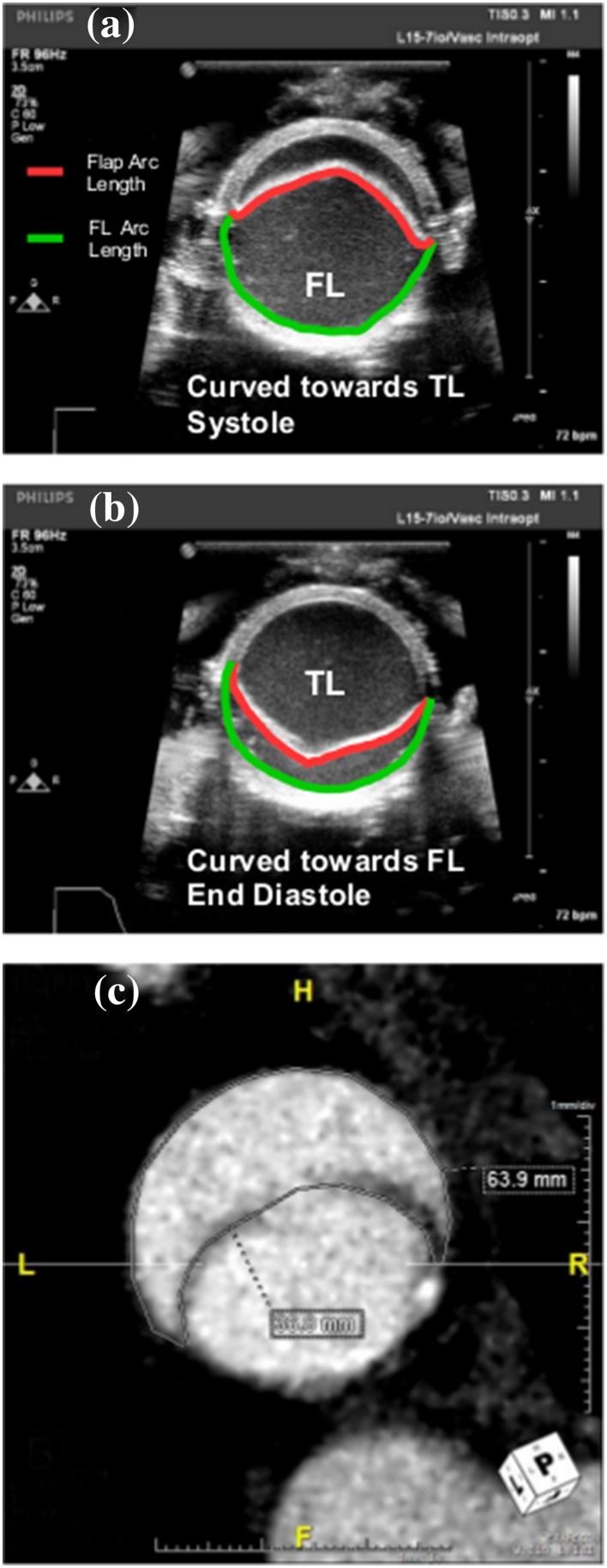
Cross sectional view of post propagated aorta vessel highlighting flap arc length and the false lumen (FL) arc length used to calculate the False lumen Dissection Ratio (FDR). (**a**) The flap curved towards the True lumen (TL) during systole phase while the flap was curved towards the False Lumen (FL) in porcine aorta as seen in (**b**). (**c**) Representative non-gated CT scanned image depicting human descending thoracic aorta with the two measurements for the circumference of the false lumen (63.9 mm) and the length of the dissection flap (36.8 mm).

To characterize the lumen cross sectional area changes post-propagation and with distal re-entry tear, the %TL CSA at peak systole and diastole pressures were assessed as described in Peelukhana *et al.* 20 To identify the extent of the false lumen expansion post propagation and with creation of a distal re-entry tear, we defined the ratio of the arc length of false lumen (dissected wall) to arc length of flap as False lumen Dissection Ratio (FDR) as shown in Fig. 2. FDR of value 1 implies no dissection (i.e., intact wall) while FDR >1 implies dissection of greater degrees with increasing value of FDR. Using Fiji image processing software,^26^ the arc length of the false and true lumen along with the flap length were measured at all locations (proximal, middle and distal) for each pulse pressure (120/80, 140/80, 160/80 mmHg). For a given location and pulse pressure, the FDR was averaged over the peak systolic and diastolic pressure. At the proximal end for a given pulse pressure, the %undissected wall was defined as the ratio of TL arc length to arc length at 0% dissection (baseline). The circumferential Green strain on the flap was quantified as:

$$\frac{1}{2}\left( {\frac{{({\text{Flap}}\; {\text{Arc}}\; {\text{Length}}_{{{\text{Peak}}\; {\text{Systole}}}}^{2} ) - ({\text{Flap}}\; {\text{Arc}}\; {\text{Length}}_{\text{Diastole}}^{2} )}}{{({\text{Flap}}\; {\text{Arc}}\; {\text{Length}}_{\text{Diastole}}^{2} )}}} \right) \times 100$$

The thicknesses of the flap along with FL and TL at proximal and distal ends were measured using a micrometer caliper (Mitutoyo America Corporation, Aurora, Il, USA) before and after the creation of a distal re-entry tear. The average of three readings was used to obtain the final value. The %change in the thickness was calculated as:

$$\left( {\frac{{{\text{False}}\; {\text{Lumen}}\; + \;{\text{Flap}} - {\text{True}}\; {\text{lumen}}\; {\text{thickness}}}}{{{\text{True}}\; {\text{lumen}}\; {\text{thickness}}}}} \right) \times 100.$$

The data were assessed for normality (skewness and kurtosis). Descriptive statistics were calculated for all the variables. Statistical analysis was two-tailed and was carried out at an alpha level of 0.05. Variables were logarithmically transformed in case of deviation from normality. Chi square and *t*-tests were used to assess significance for categorical and continuous variables respectively. Linear mixed-effects regression models were used to assess the effect of percent of undissected wall circumference and pulse pressure at a fixed location (proximal end) on FDR for porcine aortae and the effect of demographic (age, gender) and clinical variables (hypertension, systole and diastolic pressure) on patient FDR values. Repeated measures ANOVA was used to evaluate the relationship between location within the dissected portion, pulse pressure and presence of re-entry tear on the FDR values. Spearman’s rank correlation was utilized to test the strength of relationship between FDR values and gender for patient data. All data are expressed as mean (S.D).

### Clinical FDR Data

Clinical data was gathered from the Cook Medical STABLE I clinical trial which evaluated the Zenith Dissection Endovascular System in the treatment of Type B aortic dissections for comparative analysis of FDR between the *in vitro* test setup and the *in vivo* conditions. In accordance with current legislative recommendation, the interventions were performed with approval of the Institutional Review Board. All patients were informed in detail about the endovascular intervention and gave written consent. While the trial evaluated both acute and chronic aortic dissections, only those patients treated for acute, complicated Type B aortic dissection were included in the analysis. Additionally, patients who had poor or no pre-operative imaging from the trial were excluded from this analysis. For each case, non-gated computed tomography (CT) images were utilized to first identify the primary entry tear. Measurements of the dissection flap length and false lumen arc length were performed at the first slice distal to entry tear. The measured FDR per patients represents the cycle average value. An example is this measurement is depicted in Fig. 2c.

## Results

### Lumen Pressure Characteristics of Type B Dissection

The mean proximal circumferential propagation was 72.8 (5)% and the average axial length after propagation was 12.6 (1.2) cm.

**Figure 3:**
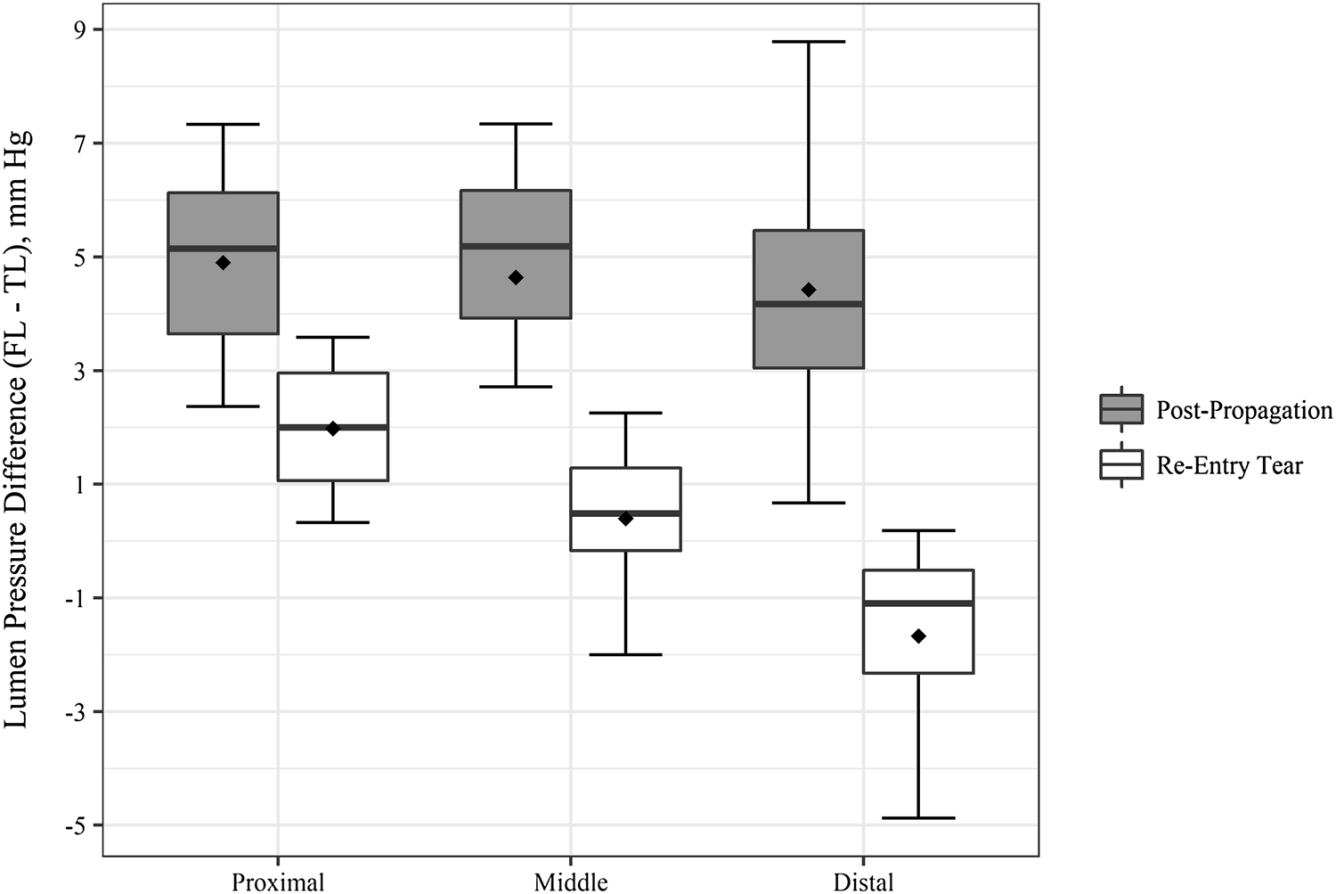
Boxplot showing the spatial variation and mean values of lumen pressure difference between FL and TL after propagation with and without distal re-entry tear. Mean values are represented by diamond symbol within the boxplot.

The magnitude of the lumen pressure difference after propagation was consistent across the three spatial locations with mean value of 4.6 (2.1) mmHg with higher pressure measured within the FL (Fig. 3). As expected, the mean pressure difference approached zero with the creation of the distal re-entry tear [0.24 (1.4) mmHg]. The longitudinal profile of the lumen pressure difference, however, indicated a gradient along the length of the dissected portion with maximum difference at the distal end [% re-entry circumference = 45.7 (6.8)].

Interestingly, the absolute magnitude of the measured pressure increased in both the lumens while the pressure difference across did not vary significantly before and after propagation. A mean percent increase in pressure magnitude of 27.9 (7)% was measured in the FL compared to 33.3 (11)% in TL. In addition to the alleviation in the lumen pressure differential, the creation of the re-entry tear also restored the pressure magnitude to that measured prior to propagation.

**Figure 4:**
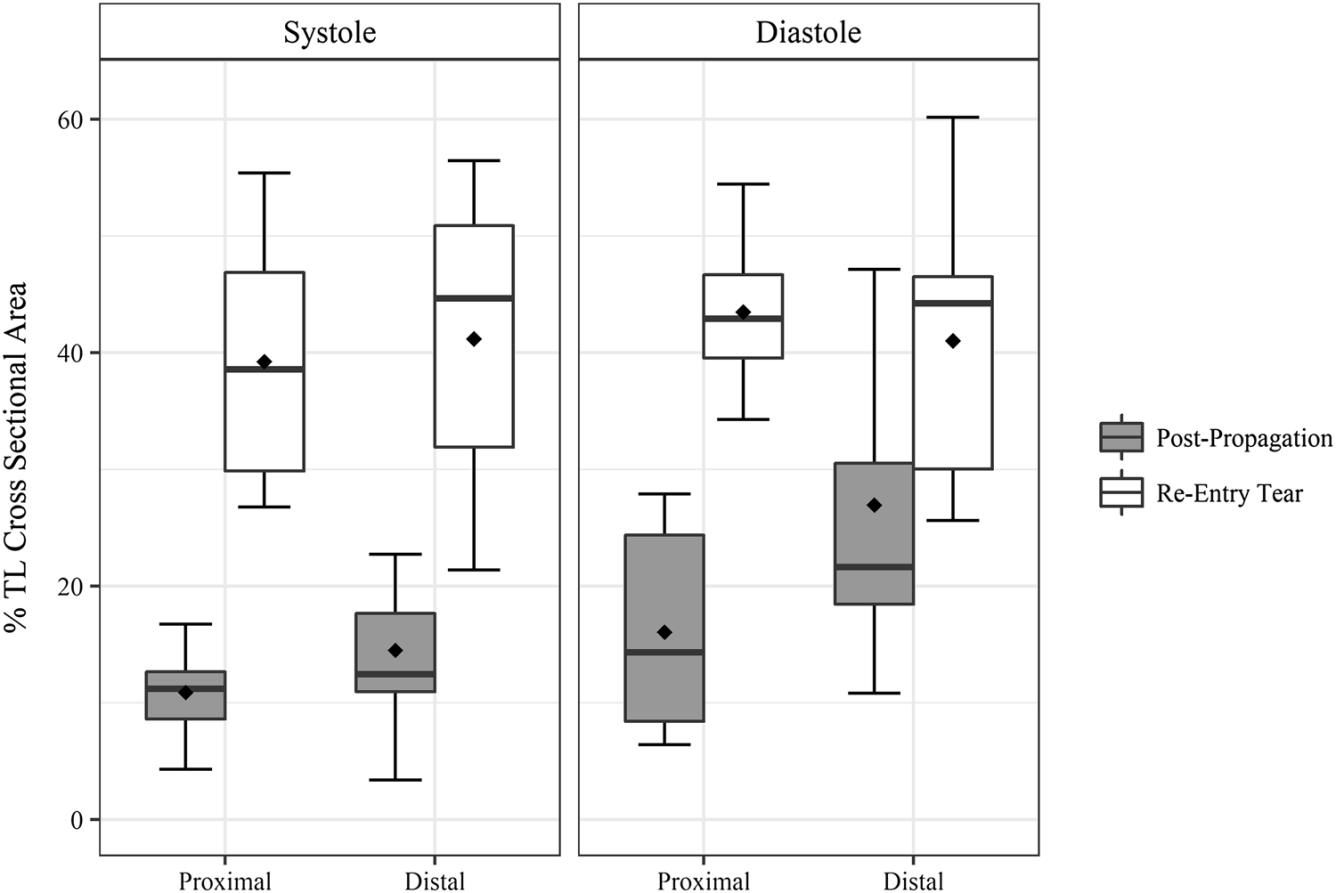
Boxplots showing the distribution and mean %TL cross sectional area of TL at peak systole and diastole without and with the creation of re-entry tear. Mean values are represented by diamond symbol within the boxplot.

Position and configuration of the intimal flap has been shown to correlate with dynamic obstruction^10^ in aortic dissection. The flap dynamics were assessed by quantifying the flap configuration and the TL cross sectional area over peak systole and diastole phase of the cardiac cycle at the proximal and distal locations within the dissected portion of the vessel. The flap configuration at the proximal end of the vessel was curved towards FL during systole phase in 37.5% of samples and doubled to 75% with re-entry tear. Similar trend was observed at the distal end of the vessel with the flap curvature towards FL in 12.5% of samples post propagation to 75% with re-entry tear. Similar to pre-propagation,^20^ the flap curvature was towards FL in diastole and was identical for proximal and distal ends increasing from 75% and 25% respectively to 100% with re-entry tear (Fig. 2). The gain in %TL CSA with the creation of the distal re-entry was significant at both proximal [*t*(15) = 12.67, *p* = 2.034*e*−09] and distal end [*t*(15) = 5.85, *p* = 3.164*e*−05] as shown in Fig. 4.

In two of the aortic vessels post propagation (excluded from analysis), the flap collapsed onto the true lumen with near complete occlusion both at proximal and distal ends. Consequently, large pressure differentials were measured between the lumens after propagation [76 (14) mmHg]. The creation of the distal re-entry tear, however, eliminated the pressure difference between the lumens.

### False Lumen Dilation Ratio (FDR) and Vessel Geometry

Ratio of TL to flap arc length was calculated to account for contribution of elastic recoil and shortening of the flap to the FDR values and was consistent across samples [1.01 (0.17)]. The depth of the dissection plane within the media which also influence the FDR and dilation, measured 35.74 (1.7)% at proximal dissection increasing to 51.26 (5.8)% at distal end of the dissection. The %change in wall thickness which assess the lateral expansion in the vessels within the experimental period, was not significant at proximal [*t*(5) = 1.65, *p* = 0.15] and distal [*t*(5) = 1.46, *p* = 0.20] portions of the dissected vessel with the creation of the re-entry tear.

**Figure 5:**
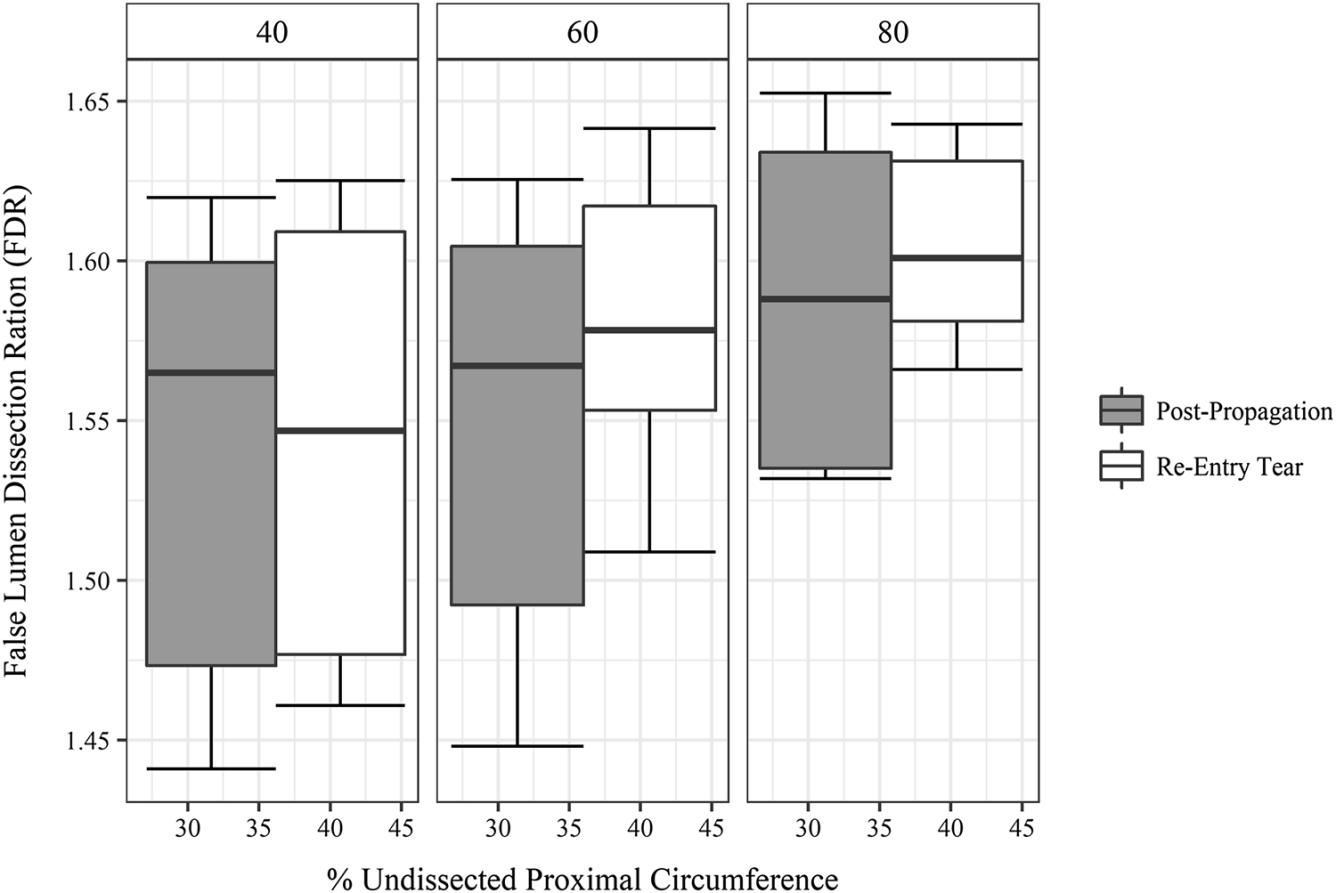
Distribution of False lumen Dissection Ratio (FDR), defined as the ratio of false lumen arc length to the arch length of the flap with %undissected wall circumference at the proximal end with increase in pulse pressure (40, 60, 80 mmHg). The diastole pressure was kept constant at 80 mmHg with increase in pulse pressure.

By using random effects for sample, depth of dissection plane and the percent circumference of TL wall, we controlled for different mean ratings associated with these variables. For a fixed location (proximal end), pulse pressure influenced FDR [*χ*(2) = 19.81, *p* = 0.00005] by increasing it by 0.026 (0.005) for every pulse pressure increase of 20 mmHg. Presence of re-entry tear increased the FDR when compared to post-propagation values by 0.008(0.004) and was significant (*p* = 0.0003). However, the percent of original wall circumference was not significant on FDR values (Fig. 5).

**Figure 6:**
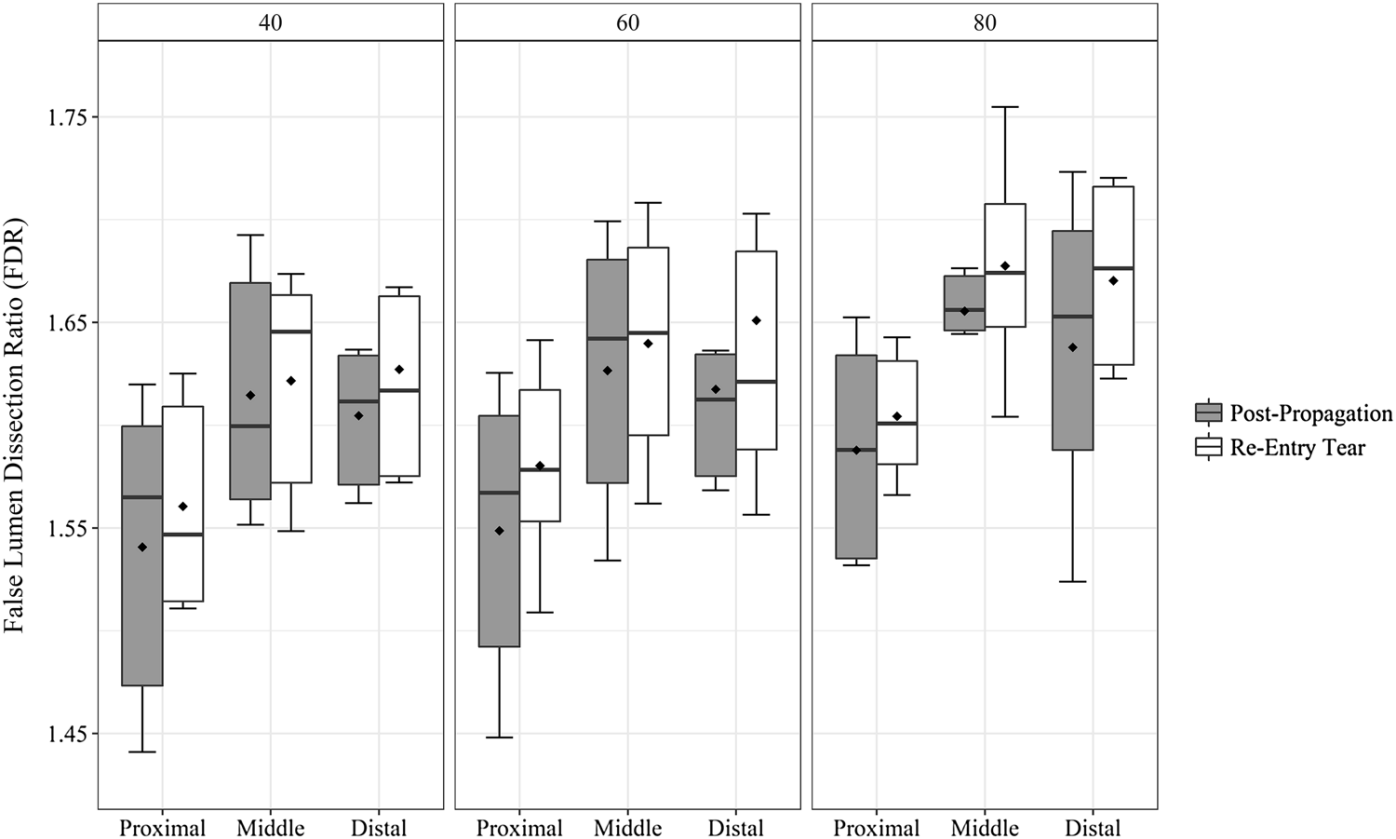
Changes in FDR values over the length of the dissection with increasing pulse pressure (40, 60, 80 mmHg). Higher values of FDR are associated with creation of the re-entry tear and distal end of the dissection. Mean values are represented by diamond symbol within the boxplot.

Presence of distal re-entry tear [*F*(1,15) = 13.89, *p* = 0.002] and location within the dissected portion of the vessel [*F*(2,30) = 5.382, *p* = 0.01] significantly influenced the FDR values, while the effect of increasing pulse pressure was not significant (Fig. 6). FDR was higher with the presence of large distal re-entry tear [1.62 (0.083)] when compared to post-propagated values [1.60 (0.085)]; a significant increase of 0.017 [95% CI, 0.008–0.026, *t*(53) = 3.848, *p* = 0.0003]. FDR values also increased by 0.072 [95% CI, 0.048–0.095, *t*(35) = 6.19, *p* = 4.3*e*−07] in the middle [1.64 (0.06)] and by 0.068 [95% CI, 0.03–0.107, *t*(35) = 3.66, *p* = 0.0008] in the distal portion [1.63 (0.11)] when compared to proximal [1.56 (0.06)] end of the dissected portion of the aortic vessel.

No new dissections were observed within any of the aortic specimens and arterial branches were not involved.

**Figure 7:**
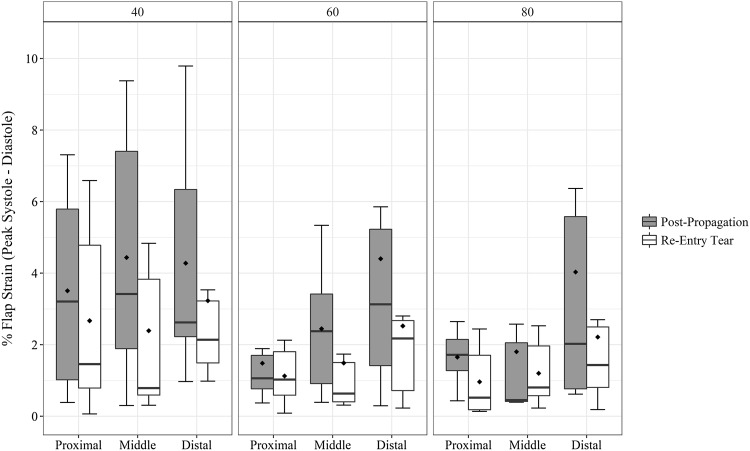
Changes in circumferential Green strain in the flap over the length of the dissected aorta with increasing pulse pressure (40, 60, 80 mmHg). Flap strain values are cycle averaged; lower values are associated with the creation of re-entry tear. Mean values are represented by diamond symbol within the boxplot.

Similar to FDR, presence of distal re-entry tear [*F*(1,15) = 5.34, *p* = 0.035] and location within the dissected portion of the vessel [*F*(2,30) = 6.23, *p* = 0.005] significantly influenced the circumferential flap strain (Fig. 7). The increasing pulse pressure did not influence the flap strain values. Lower values of flap strain were associated with the creation of distal reentry tear [*t*(53) = 2.72, *p* = 0.008]. The circumferential flap strain increased significantly along the length of the vessel with maximum values at distal end when compared to proximal [*t*(35) = 2.67, *p* = 0.011] and middle [*t*(35) = 3.21, *p* = 0.003] portions.

### Demographic Summary and Clinical FDR Measurements

Thirty patients [21 men (70%) and 9 women (30%)] with average age of 57.9 (11.9) years were included in this study. Twenty-five (83.3%) of the patients presented as hypertensive at the time of diagnosis, with average pressures 141.5 (42.6) mmHg and 75.5 (26.6) mmHg for systolic and diastolic respectively and none were diabetic. Amongst this patient set, 20 (58%) had multiple entry tears and 18 (52%) had more than two re-entry tears. Detailed clinical summary is listed in Table 1.

**Table 1:** Demographic and Clinical summary of patients with acute Type B dissection in STABLE I trial.

Patient ID	FL Circ at entry (mm)	Flap Length at 1st Communication (mm)	FDR	Age (years)	Sex	Hypertension	Systolic blood pressure (mmHg)	Diastolic blood pressure (mmHg)
1	48.4	25	1.94	50	Female	Yes	119	54
2	60.7	39.8	1.53	62	Female	Yes	100	60
4	62.2	33.1	1.88	73	Female	Yes	145	74
5	63.9	36.8	1.74	72	Female	Yes	142	59
6	65.8	35.4	1.86	49	Female	Yes	150	81
7	74	28.6	2.59	31	Female	No	140	80
8	78.6	45	1.75	56	Female	Yes	130	60
9	89.7	35.8	2.51	61	Female	Yes	100	70
10	121	54.9	2.20	78	Female	No	124	80
11	62.8	34.1	1.84	45	Male	Yes	116	96
12	62.9	41.9	1.50	47	Male	Yes		
13	63.9	31.4	2.04	37	Male	No	159	96
14	65.4	45.8	1.43	79	Male	Yes	190	90
15	66.1	34.5	1.92	57	Male	Yes	165	89
16	66.6	40.6	1.64	65	Male	Yes	176	92
17	66.7	41.3	1.62	55	Male	Yes	152	59
18	68.2	33.7	2.02	50	Male	Yes	255	150
19	76	40.5	1.88	61	Male	Yes	150	100
20	76.3	38.8	1.97	67	Male	Yes	142	68
21	77.8	38	2.05	63	Male	No	136	53
22	78.3	45	1.74	60	Male	Yes	118	60
23	81.8	40.7	2.01	74	Male	Yes	130	60
24	84	52.6	1.60	68	Male	Yes	127	58
25	90.2	39.8	2.27	49	Male	Yes	130	57
26	91.8	45	2.04	55	Male	Yes	230	130
27	92.6	62.3	1.49	42	Male	Yes	127	90
28	93.4	50.1	1.86	52	Male	Yes	130	67
29	95.2	66.8	1.43	60	Male	Yes	150	80
30	106	51.5	2.06	49	Male	No	142	83
31	107	56.9	1.88	71	Male	Yes	170	70

The FDR for the entire dissection cohort measured 1.87 ± 0.27, with no statistical differences between men and women. Hypertension significantly influenced FDR values after controlling for age and gender (*χ*(1) = 8.11, *p* = 0.0044) with decrease by 0.36 (0.12) when compared to subjects without hypertension. Systolic and diastolic pressure values did not influence the measured FDR values for these subjects.

Significant correlation was observed between sex and the flap length at first communication (*ρ* = − 0.43, *p* = 0.023) with higher values measured for males when compared to females. The minimum FDR for the entire cohort was 1.43 and the maximum FDR was 2.59, thus showing that in all acute dissection patients contraction of the dissection flap occurs in combination with expansion of the separated false vessel wall and significant extension is needed to re-appose the dissection flap to the false lumen wall.

## Discussion

Endovascular treatment using stent-graft to treat aortic Type B dissection has produced acceptable outcome.^7^,^8^,^18^ The relatively high incidence of increasing aortic diameter, however, require subsequent intervention. These considerations make it important to understand the physical properties of the aortic wall and the influence of these properties on aortic wall behavior after acute aortic dissection. In this reproducible *ex vivo* model of Type B acute aortic dissection, the magnitude of the lumen pressure difference was minimal post propagation, dependent on flap movement, and was restored to equilibrium with the creation of distal re-entry tear. Large dilation of the false lumen (FDR >1.6) was measured post-propagation that was dependent on regional biomechanical properties of the aorta, independent of pulse pressure and local lumen pressure differences, and was not restored with the creation of distal re-entry tear. Interestingly patients with acute Type B dissection had overall higher mean FDR values (~ 1.88) with higher values for normotensive patients independent of age and sex. These results highlight the requisite for significant extension required to re-appose the flap against the false lumen wall as well as consideration for the mechanical properties of the aortic wall for successful intervention.

The true lumen compression was attributed to greater pressure in false lumen and due to pulsatile dynamics of the flap. As evident from the changes in true lumen cross sectional area, a discrepancy in lumen sizes did not imply the existence of a pressure gradient. The transmural pressure showed dependence on local anatomical biomechanical properties evident from the longitudinal lumen pressure distribution along the length of the dissection and was more pronounced with the presence of re-entry tear. Although the gain in the cross-sectional area of the true lumen along the dissection length was significant with the creation of the distal re-entry tear, it did not re-expand to baseline due to the elastic recoil of the dissection flap. Clinically, intravascular stents are deployed to expand the true lumen and may result in tearing or rupture to the flap since the deployment of stent does not alter the pressure gradient across the flap. Although the measured magnitude of the pressure difference post-propagation was small (~ 5 mmHg), the difference was positive over the entire cardiac cycle (peak systolic, diastolic and mean pressure). Previous *in vitro* models of aortic dissection using phantom materials showed decreased systolic pressure instead of significant increase in the diastolic pressure within the false lumen in the absence of an exit tear as compared to the true lumen.^1^,^25^,^35^ This could be attributed to a mismatch between the shear modulus and the tensile strength of the polymer phantom compared to aortic tissue along with presence of a rigid flap.

Relevance of an *ex vivo* model of dissection is related to reproducibility of the characteristics of human Type B dissection. Following dissection, the overall aortic dilation was due to the false lumen expansion (increasing FDR values). The true lumen circumference which is flanked by dissection flap at negligible transmural pressure and original elastic rich wall remained relatively unchanged before and after creation of the re-entry tear. Patency of the false lumen is identified as an independent clinical risk factor to predict aortic dilation^15^,^28^,^33^ with increased false lumen pressure due to outflow restrictions (partial thrombosis or uneven tear size) as explanation for aortic dilation.^1^,^35^ In the absence of trans dissection flap pressure gradient it is important to evaluate the load on the flap and the FL wall considering current treatment options i.e., stent graft or septectomy. The uneven distribution of the elastin layers in the false lumen after the partial separation of the media layers reduces the compliance of the aortic wall. Subsequently the circumferential stress in the vessel wall which is uniform under physiological conditions will be altered.^12^ In addition regional anatomy dictates variation in nonlinear biomechanical response due to changes in wall thickness and collagen content with blood pressure.^11^,^14^,^37^

For a fixed location within the dissection length, increasing pulse pressure and patent false lumen led to increased FDR values for a consistent depth of dissection. Pulse pressure was however not a critical factor for the increased dilation of the false lumen along the entire length of the dissection. Local regional properties evident from changes in FDR values between the proximal to distal end of the aorta and the presence of a large distal tear were overall significant factors influencing false lumen dilation. These results suggest a dissociation between pressure gradient and risk for dilation. The false lumen dilation increases relative to flap i.e., increase in FDR but the flap dimensions in the circumferential and lateral directions do not change significantly with increase in pulse pressure indicating a structural limitation to expansion. Significant stiffness of the aged human aorta when compared to porcine aorta or lack of completely developed distal tears could explain the higher measured values of FDR in human patients with Type B dissection.^16^,^33^ While the significant effect of hypertension on FDR values was anticipated; association of higher FDR values with normotensive patients was surprising and is not readily explainable. This may have resulted due to the small sample size, inclusion of younger patients in the cohort or variation in the initiation of the entry tear between subjects. Although we did not observe any compensating change in both true and false wall thickness over the short (~h) experimental timeline, long term *in vivo* remodeling may influence the stress distribution and further remodeling.^7^,^17^,^18^ It is also important to note that current endovascular treatments that target re-apposition of the aortic wall are not designed for complete compensation of the initial dilation of the wall.^18^ This results in a patent false lumen (below the level of thoracic stent graft) which over time increases the need for intervention.^15^

Some limitations of the current study should be acknowledged. First the effect of aortic arch anatomy was not accounted for during the experiments. Second, the results of this study pertain only to acute dissections in the absence of false lumen remodeling. Third, neo-intimal thickening of the flap has been reported clinically during follow-up, which might restrict the flap movement. Fourth, we replicated dissections with two tears while multiple tears are regularly observed during clinical imaging.^22^ Fifth, the tears were perpendicular to the flow which may not represent the biologically relevant orientation; i.e., spiral tears have been reported.^4^ Sixth, the FDR and static pressure measured likely depend on size of the tear. Finally, effects of patent side branches which has been shown to increase the false lumen cross sectional area^36^ were not accounted and warrant further investigation.

## Conclusions

Higher FDR values (FDR = 1 implies zero dissection) in the presence of distal re-entry tear demonstrate an acute change in vessel morphology in response to the dissection independent of local pressure. This retraction of the flap is likely a result of residual strain in the wall and makes re-apposition of the aortic wall more challenging.
